# Supplementary material for: Content-rich biological network constructed by mining PubMed abstracts
Source: BMC Bioinformatics. 2004 Oct 8;5:147. doi: 10.1186/1471-2105-5-147 (PMC528731; doi:10.1186/1471-2105-5-147)
Supplement: Additional File 2 — The original results of the above study (non-essential files are deleted to keep the file size under the limit set by BMC bioinformatics). [file 1471-2105-5-147-S2.bz2 › chilibotAdditionalFile2/dip10/59ID8414517E214/html/RBBP2_RB1.html]

 


 **RBBP2** and **RB1** 
  
Found 11 abstracts in PubMed, retrieved 10.  
 

 What does Google say? 
 PDF only 
| .edu only 

---

**Interactive relationship** (e.g. stimulation, inhibition, etc)

**Neutral relationship**- The NH 2 terminal end of a protein, named SMCp, which contains an ARID A T rich interaction domain DNA binding domain and is similar to the mammalian SMCY SMCX proteins and retinoblastoma  [ **RB1** ]  binding protein 2  [ **RBBP2** ] , was shown to bind the African swine fever virus encoded ubiquitin conjugating enzyme UBCv1 using the yeast two hybrid system and in in vitro binding assays.  Ref: 10760505 FEBS Lett, 2000
- Retinoblastoma  [ **RB1** ]  binding protein 2  [ **RBBP2** ]  Rbp2 was originally identified as a retinoblastoma  [ **RB1** ]  protein RB pocket domain binding protein.  Ref: 11358960 J Biol Chem, 2001

**Non-interactive relationship** (e.g. studied together, co-existance, homology, etc.)

- Using the RBTN2 LIM domain region as bait, the retinoblastoma  [ **RB1** ]  binding protein 2  [ **RBBP2** ]  RBP2 was identified as a partner for RBTN2.  Ref: 9129143 Oncogene, 1997
- Isolation and chromosomal localization of a new human retinoblastoma  [ **RB1** ]  binding protein 2  [ **RBBP2** ]  homologue 1a RBBP2H1A.  Ref: 10878660 Eur J Hum Genet, 2000
- these genes encode very diverse proteins, including signaling molecules such as BMP 4, the enzyme superoxide dismutase, the cell adhesion molecule cadherin 6, proteins involved in gene transcription such as HMG 1 and SAP18, homeodomain containing proteins Gbx 2 and Evx 2, and cell cycle regulatory proteins such as the retinoblastoma  [ **RB1** ]  binding protein 2  [ **RBBP2** ] .  Ref: 10942599 Exp Cell Res, 2000
- A screen for new trithorax group genes identified little imaginal discs, the Drosophila melanogaster homologue of human retinoblastoma  [ **RB1** ]  binding protein 2  [ **RBBP2** ] .  Ref: 11014813 Genetics, 2000
- Of the remaining 76 cDNA sequences, 11 matched previously identified human chromosome 12p genes and 3 matched previously determined cDNA sequences, including the retinoblastoma  [ **RB1** ]  binding protein 2  [ **RBBP2** ]   **RBBP2** , the cyclin dependent kinase inhibitor KIP1, and an expressed sequence tag.  Ref: 8530100 Genomics, 1995
- Among the partial cDNAs cloned, a novel cDNA was identified, which showed 54% identity to the recently cloned cDNA of the retinoblastoma  [ **RB1** ]  binding protein 2  [ **RBBP2** ]  RBP2.  Ref: 10616211 Lab Invest, 1999
- A protein with similarity to the human retinoblastoma  [ **RB1** ]  binding protein 2  [ **RBBP2** ]  acts specifically as a repressor for genes regulated by the b mating type locus in Ustilago maydis.  Ref: 11029697 Mol Microbiol, 2000
- T cell oncogene rhombotin 2 interacts with retinoblastoma  [ **RB1** ]  binding protein 2  [ **RBBP2** ] .  Ref: 9129143 Oncogene, 1997
- Deficiency of a novel retinoblastoma  [ **RB1** ]  binding protein 2  [ **RBBP2** ]  homolog is a consistent feature of sporadic human melanoma skin cancer.  Ref: 10616211 Lab Invest, 1999
- Retinoblastoma  [ **RB1** ]  binding protein 2  [ **RBBP2** ]  Rbp2 potentiates nuclear hormone receptor mediated transcription.  Ref: 11358960 J Biol Chem, 2001
- The protein encoded by the little imaginal discs gene is the Drosophila homologue of human retinoblastoma  [ **RB1** ]  binding protein 2  [ **RBBP2** ] .  Ref: 11014813 Genetics, 2000
- Using a NotI linking clone NR 025 as a probe, we isolated a novel putative member of the RB binding protein family, namely a human retinoblastoma  [ **RB1** ]  binding protein 2  [ **RBBP2** ]  homologue RBBP2H1A.  Ref: 10878660 Eur J Hum Genet, 2000
- These include human hairless, mutated in individuals with alopecia universalis, retinoblastoma  [ **RB1** ]  binding protein 2  [ **RBBP2** ]  and several putative chromatin associated proteins.  Ref: 11165500 Trends Biochem Sci, 2001
- In a screen for components of the b dependent regulatory cascade we have isolated Rum1 regulator U. maydis 1, a protein with similarities to the human retinoblastoma  [ **RB1** ]  binding protein 2  [ **RBBP2** ] .  Ref: 11029697 Mol Microbiol, 2000
